# Human papillomavirus vaccination in adults: impact, opportunities and challenges – a meeting report

**DOI:** 10.1186/s12919-021-00217-4

**Published:** 2021-08-12

**Authors:** Dur-e-Nayab Waheed, John Schiller, Margaret Stanley, Eduardo L. Franco, Mario Poljak, Susanne K. Kjaer, Marta del Pino, Fiona van der Klis, Maarten F. Schim van der Loeff, Marc Baay, Pierre Van Damme, Alex Vorsters

**Affiliations:** 1 Centre for Evaluation of Vaccination, Vaccine & Infectious Disease Institute, University of Antwerp, Antwerp, Belgium; 2 Center for Cancer Research National Cancer Institute, Bethesda, MD 20814 USA; 3 Division of Cellular and Molecular Pathology, University of Cambridge, Cambridge, UK; 4 Division of Cancer Epidemiology, McGill University, Montreal, Quebec Canada; 5 Institute of Microbiology and Immunology, Faculty of Medicine, University of Ljubljana, Ljubljana, Slovenia; 6 Danish Cancer Society Research Center, Unit of Virus, Lifestyle and Genes, and Department of Gynecology, Rigshospitalet, University of Copenhagen, Copenhagen, Denmark; 7 Gynecology Oncology Unit. Institute Clinic of Gynecology, Obstetrics, and Neonatology (ICGON), Hospital Clínic of Barcelona. Institut d’Investigacions Biomèdiques August Pi i Sunyer (IDIBAPS), Universitat de Barcelona, Barcelona, Spain; 8 National Institute for Public Health and the Environment (RIVM) | RIVM and Centre for Infectious Disease Control (CIb), Utrecht, Netherlands; 9 Department of Infectious Diseases, Public Health Service (GGD) Amsterdam, and Amsterdam UMC, and University of Amsterdam, and Internal Medicine, Amsterdam institute for Infection and Immunity (AII), and Amsterdam Public Health Research Institute, Amsterdam, Netherlands; 10 P95, Epidemiology and Pharmacovigilance Consulting and Services, Leuven, Belgium

**Keywords:** Human papillomavirus, HPV vaccine, Vaccination, Adults

## Abstract

For more than a decade human papillomavirus (HPV) vaccine have been implemented in most high-income countries, and more recently also in several low- and middle-income countries. The vaccines are safe and their impact and effectiveness in preventing HPV vaccine type infection and associated diseases has been thoroughly established. Currently, the primary recommended cohorts for immunisation are adolescents, 9–15 years of age but HPV is an ubiquitous infection that is mainly (but not exclusively) sexually transmitted. Sexually active adults remain susceptible to infection and continued transmission of the virus, representing a reservoir of infection in the population. A recent meeting, conducted by the HPV Prevention and Control Board (HPV-PCB), reviewed the current status of HPV vaccination of adults, discussed limitations, challenges and benefits of HPV vaccination of adults, evaluated the effectiveness of HPV vaccination after treatment of post cervical cancer and precancerous lesions, and discussed the potential impact of adult vaccination on cervical cancer elimination strategies in light of the current and future HPV vaccine shortage. HPV-PCB is an independent multidisciplinary board of international experts that disseminates relevant information on HPV to a broad array of stakeholders and provides guidance on strategic, technical and policy issues in the implementation of HPV prevention and control programs. The HPV-PCB concluded that, given the current data available on adult HPV vaccination and the ongoing vaccine supply constraints, it is too early to implement routine vaccination of adults. Many research gaps need to be filled before we have a better understanding of the efficacy and broader public health impact of HPV vaccination in adult women.

## Background

The Human Papillomavirus (HPV) Prevention and Control Board (www.hpvboard.org) (HPV-PCB) is an international independent multidisciplinary board that was created in 2015 [1]. The HPV-PCB is a group of experts who provide evidence-based guidance on strategic, technical and policy issues that occur as part of the implementation of HPV control programmes. The HPV-PCB aims to generate and disseminate relevant information on prevention and control of HPV-associated diseases to a broad array of stakeholders. It achieves its objectives by organizing two meetings per year [1–5]. One is a technical meeting covering topics such as vaccine efficacy, vaccine safety, screening technologies and policies, treatment strategies, and approaches to address vaccine hesitancy. The second meeting is a country-specific meeting covering a Strength, Weakness, Opportunities and Threats (SWOT) analysis of a country or region. This report covers the seventh meeting of the HPV-PCB held in Antwerp, Belgium (12–13 November 2019): a technical meeting on challenges, impact and opportunities for HPV vaccination in adults.

HPV vaccines have been implemented in the National Immunization Programmes (NIPs) of several high-income countries (HICs) for 10 years or more. These vaccines are safe, and their impact and effectiveness in preventing vaccine-type HPV infection and associated diseases are scientifically confirmed. Currently, the recommended cohorts for immunisation are adolescents 9–15 years of age, since HPV vaccines are prophylactic and confer protection to infection before the onset of sexual activity. Unvaccinated sexually active adults remain susceptible to infection and contribute to transmission, thus representing a reservoir of infection in the population. Immunisation of older cohorts could potentially restrict HPV transmission and thus contribute to reducing infection prevalence, which would accelerate the population impact of these vaccines on both benign and malignant HPV-associated diseases. However, there is limited evidence to support these assertions and the question is what the magnitude of this transmission might be and how cost-effective vaccination would it be to prevent it. The meeting was held to address the following issues:

- To provide an overview of the current status of HPV vaccination in adults.
- To review the immunogenicity, safety and efficacy data from existing studies on HPV vaccination in adult women.
- To gain insight into the efficacy of the HPV vaccine at the mucosal and systemic levels
- To discuss ways and methods to conduct effective research on the potential benefits of vaccinating adults likely to have prior genital exposure to HPV
- To discuss challenges and benefits of vaccination in adults, including high-risk groups.
- To discuss cervical cancer elimination strategies and the impact of adult vaccination on elimination strategies
- To discuss the potential implication of vaccination in adults on vaccine supplies in low- and middle-income countries (LMICs)

This report summarizes the discussions and lessons learned from the participants.

## Situational analysis of HPV vaccination in adults: immunogenicity and safety data

### Efficacy and safety of prophylactic HPV vaccination in adults

The results of the recent Cochrane review by Arbyn et al. were discussed [6]. The review evaluated the risks and benefits of prophylactic HPV vaccines against cervical precancer lesions and HPV-16/18 infection in adolescent girls and women, including randomised controlled trials (RCTs) comparing efficacy and safety in females offered an HPV vaccine or placebo (vaccine adjuvants or another control vaccine). The study included data from 26 trials, of which three recruited women aged 25 and over. The effects of the vaccines in participants who had at least one vaccine dose were summarized [6].

There is high-certainty evidence that HPV vaccines protect against cervical precancer lesions in adolescent girls and young women aged 15 to 26 years. The effect is higher for lesions associated with HPV-16/18 than for lesions irrespective of the HPV type. The effect is greater in those who are negative for high risk HPV (hr)HPV) or HPV-16/18 DNA at enrolment than in those unselected for the HPV DNA status. There is moderate-certainty evidence that HPV vaccines reduce the incidence of cervical intraepithelial neoplasia (CIN) grade 2+ in older women who are HPV-16/18-negative but not when they are unselected by the HPV DNA status [6]. Similarly, in a nested case-control study of women enrolled in Kaiser Permanente Northern California with women with CIN2+ or CIN3+ as cases and age-matched women without CIN2+ or CIN3+ as controls, the strongest protection against CIN2+ was found in women who had received their first dose between the age of 14 and 17 years (relative risk (RR) 0.52, 95% confidence interval (CI) 0.36–0.74) or 18 and 20 years (RR 0.65, 95% CI 0.49–0.88) [7]. No significant protection was found in women aged 21 years or older at their first dose (RR 0.94, 95% CI 0.81–1.09). Similar results were obtained for CIN3+ with the first dose at ages 14–17 years (RR 0.27, 95%CI 0.13–0.56) or ages 18–20 years (0.59, 95%CI 0.36–0.97), leading to the conclusion that catch-up quadrivalent HPV vaccination with quadrivalent vaccine was effective against CIN2+ and CIN3+ in girls and women aged 14–20 years at the time of the first vaccine dose but not for women aged 21 years and older at the first dose [7].

Based on four RCTs in adult women, two with the bivalent vaccine and two with the quadrivalent vaccine, no increased risk of serious adverse effects (RR = 1.05, 95% CI 0.91–1.21) was observed [6].

### Immunogenicity and tolerability of the HPV vaccine in women aged 15–55 years

A long-term follow-up study (NCT00947115) of females aged between 15 and 55 years at first vaccination with the bivalent HPV vaccine was discussed [8]. Participants in the primary phase III study (NCT00196937) were invited to attend annual evaluations of long-term immunogenicity and safety. Anti-HPV-16/18 antibodies in serum and cervicovaginal secretions (CVS) were measured using an enzyme-linked immunosorbent assay (ELISA). Seropositivity rates for anti-HPV-16 remained high (≥96.3%) in all age groups 10 years after the first vaccination, whereas seropositivity for anti-HPV-18 decreased from 99.2% in 15 to 25 year olds to 93.7 and 83.8% in 26 to 45 year olds and 45 to 55 year olds, respectively [8]. Nevertheless, geometric mean titres remained above natural infection levels in all age groups: anti-HPV-16 and anti-HPV-18 titres were at least 5.3-fold and 3.1-fold higher than titres observed after natural infection, respectively, and were predicted to persist above natural infection levels for at least 30 years in all age groups [8].

Correlation coefficients for antibody titres in serum and CVS were 0.64 (anti-HPV-16) and 0.38 (anti-HPV-18), suggesting the transudation of antibodies to the cervical epithelium.

Serious adverse events (SAEs) were recorded throughout the follow-up period. Two deaths occurred in the vaccinated cohort, and both were considered not to be vaccine related [8].

It was concluded that vaccination in females aged 15–55 years elicited sustained immunogenicity with an acceptable safety profile up to 10 years after the primary vaccination, suggesting long-term protection against HPV.

A head-to-head study (NCT00423046) of three doses of the bivalent and quadrivalent HPV vaccines in healthy women aged 18–45 years showed that 5 years after vaccination, the serum neutralizing antibody responses induced by the bivalent vaccine remained 7.8-fold (18–26-years stratum), 5.6-fold (27–35-years stratum) and 2.3-fold (36–45-years stratum) higher than those induced by the quadrivalent vaccine for HPV-16. For HPV-18, the fold differences were 12.1, 13.0 and 7.8, respectively [9].

## HPV immunological dynamics at the mucosal and systemic levels

### Immune crosstalk of the HPV-specific antibody response: cross-reactivity, neutralizing activity, mucosal secretion and infection prevention

To investigate antibody seroprevalences of 7 hr-HPV genotypes (HPVs 16, 18, 31, 33, 45, 52, and 58) in two sero-surveys among the Dutch general population in the pre-vaccination era, serum samples of men and women (0–79 years of age; 1995–96 *n* =  3303; 2006–07 *n*  =  6384) were tested for anti-HPV-specific antibodies in a virus-like particle (VLP)-based multiplex immunoassay [10]. A higher overall seroprevalence in individuals older than 15 years of age was found for HPV 16, 18, 31 and 45 in 2006–07 than 1995–96. Seropositivity for one or more HPV types (2006–07, 23.1%; 1995–96, 20.0%; *p*  =  0.013) and multi-seropositivity (2006–07, 10.2%; 1995–96, 7.1%; *p* < 0.0001) increased [10]. The observed increase in specific HPV-16 seroprevalence was most likely due to changes in sexual behaviour, especially in the age of sexual debut.

Data were also shown that extended the study to a new sero-survey performed in 2016, 6 years after the introduction of the bivalent HPV vaccine to the National Immunization Programme in the Netherlands. After adjusting for demographic characteristics and sexual risk factors, a consistently lower seroprevalence of HPV16 in men (adjusted prevalence ratio (PR) = 0.7, 95% CI 0.5–0.9) was shown. In contrast, a higher seroprevalence was observed in females for HPV16 (1.3; 1.0–1.6), HPV18 (1.8; 1.3–2.3) and any HPV type (1.2; 1.0–1.3) [11]. Nevertheless, a large part of the population was seronegative. At 12 and 24 months, after the vaccination, Immunoglobulin G (IgG) antibodies could be detected in cervical secretions against HPV types 16, 18, 31, 33, 52 and 58. The correlation coefficients for antibodies in serum and CVS at 12 months were 0.58 for HPV-16 and 0.50 for HPV-18 [12].

Humoral and cellular immune responses were evaluated after different doses of the bivalent (2v) HPV vaccine in girls [13]. Blood was collected annually until 7 years post-vaccination with one, two or three doses of the vaccine (*N* = 890). HPV-type-specific IgG and Immunoglobulin (IgA) antibody levels, IgG isotypes and avidity indexes were measured by a VLP-based multiplex immunoassay for two vaccine and five non-vaccine HPV types. HPV-type-specific memory B cell numbers and T cell cytokine responses were determined in a subpopulation. HPV type-specific antibody concentrations were significantly lower in girls vaccinated with one dose than in girls vaccinated with two or three doses but remained stable over 7 years. The lower antibody response coincided with reduced HPV type-specific B and T cell responses. There were no differences in either the IgG subtypes or the avidity of the HPV-16-specific antibodies between the groups [13]. Differences between immune responses generate by the bivalent and quadrivalent HPV vaccines have been reported [14]. Currently, a study is ongoing to look at early differences in immune responses between bivalent and nonvalent HPV vaccines. Adult women (*n* = 20, 23–46 years of age, seronegative for HPV types 16, 18, 31, 33, 45, 52 and 58) were vaccinated at baseline, month 2 and month 6, and blood samples were taken at baseline and days 1, 2, 3, 6, 7, 10, and 14, 6 months, 6 months + 1 day, 6 months + 3 days, 6 months + 7 days, and 6 months + 28 days. These samples were subjected to an in-depth analysis of up to 250 innate and adaptive immune cell subsets with high-throughput flow cytometry [15], specific antibody levels were analysed by a VLP-based multiplex immunoassay and specific memory B and T cell responses were determined by enzyme-linked immune absorbent spot (ELISpot) assays. Preliminary results revealed the early expansion of several innate cell subsets at day 1. This expansion was followed by a T cell response at day 3. All donors showed a clear expansion of plasma cells, which were mostly of the IgG1 isotype, at day 7 post-vaccination. Further studies are needed to investigate whether early immune responses predict long-term antibody levels.

### Vaccine-induced HPV-specific antibodies in CVS

Because HPV infects the cervical mucosal epithelium, measuring cervical immunity is important for evaluating local immune responses to HPV infection and vaccination. Neutralizing antibodies are presumably the main effectors of protection against HPV infection and prevent the initial entry of the virus into basal epithelial cells. Humoural responses are most frequently detected in serum, whereas immune responses in CVS are usually not investigated.

Measurements of local immune responses at the cervix have been hampered by the difficulty in reliably collecting female genital secretions for immunological evaluations and in standardizing sampling to assess the variation in levels of total transudated IgG in the secretion across the menstrual cycle. Most CVS were collected by cervicovaginal lavage, during which the vaginal vault was rinsed with washing buffer. This results in sample dilution, which may complicate antibody detection. Alternatively, cervicovaginal wicks are also frequently used. These sponges, which are inserted in the vagina, passively absorb CVS. However, the application of wicks may result in microtrauma, potentially causing blood contamination and making the samples unsuitable for the detection of mucosal antibodies [16]. Alternatives for collection might be a soft cup [17] or the use of first-void urine [18].

It was concluded that antibody detection of anti-HPV antibody in CVS is feasible, although the concentration is considerably lower than that of systemic antibodies in blood. However, the high variability and the lack of a strictly uniform, well-validated method for the collection, sample preparation, detection and quantification of anti-HPV-specific antibodies at the cervix indicate the need for specific methods that can improve and standardize detection. Furthermore, heterogeneity in absorbed and extracted sample volumes requires normalization to allow valid inter-individual comparisons [16].

Most studies showed a moderate to strong correlation between anti-HPV-16/18 antibody levels in serum and CVS, indicating that vaccine-induced anti-HPV antibodies transudate from blood vessels to the cervical mucosa. The key question remains whether these transudated antibodies are needed to protect against transmission or whether exudation of systemic antibodies from blood at the sites of trauma where infections are initiated (discussed below) are sufficient. Studies are needed to clarify this point.

## Challenges and potential benefits of HPV vaccination in adults

### Pap smear collection: an increased risk of HPV infection? An objective study in a rhesus macaque model

Many features of HPV infection are still poorly understood. Productive papillomavirus infection is species- and tissue-restricted, and traditional models use animal papillomaviruses that infect the skin or oral mucosa. A mouse model of cervicovaginal infection with HPV16 was developed that recapitulates the establishment phase of papillomavirus infection. However, for infection to occur, disruption of the integrity of the stratified or columnar genital epithelium, either via chemical disruption or Cytobrush treatment, was required [19]. This led to the hypothesis that Pap smear collection, which also breaches the epithelium, may facilitate dissemination of HPV infection. This was tested in female rhesus macaques by performing a speculum examination with or without a cytology specimen collection procedure using a plastic spatula [20]. An internal digital examination was performed after specimen collection using Surgilube or carrageenan, a previously identified HPV inhibitor [21], as lubricant. The substantial infection of the ectocervix, the transformation zone, and the endocervix was detected, but only in conjunction with the cytology specimen collection procedure (1000 times higher infection rate than the control). When carrageenan gel was used as a lubricant for an internal digital examination, the number of infectious events decreased by approximately 95%. This suggests that cytology screening in women might lead to a transient enhancement of susceptibility to HPV infection and that the use of a carrageenan-based gel during the examination might mitigate this enhancement [20]. However, it was concluded that current cervical cytology sampling procedures are justified [22] because the wide use of organized screening programmes has decreased the rates of cervical squamous cell carcinoma, although not of the less frequent cervical adenocarcinomas.

The efficacy of carrageenan in reducing the risk of genital HPV infections is currently being studied in clinical trials, e.g., the LIMIT-HPV study (NCT02354144). An interim analysis of a randomized, double-blind, placebo-controlled, phase 2B trial in women (ISRCTN96104919) was recently published [23]. After a median follow-up time of 9.2 months, a total of 59 (42%) of 139 women in the carrageenan arm and 78 (57%) of 138 women in the placebo arm became DNA positive for at least one new HPV type (hazard ratio (HR) = 0.64, 95% CI = 0.45–0.89, p 0.009) [23].

### HPV vaccine post-treatment: a pathway to prevent disease relapse

A population-based cohort study conducted in Sweden between 1958 and 2008 showed that women previously diagnosed and treated for CIN3 had an increased risk of death from invasive cervical or vaginal cancer compared with the general female population [24]. Similarly, the Study of the Prevention of Anal Cancer, a 3-year prospective study of the natural history of anal HPV infection in men who have sex with men (MSM) who are at least 35 years old, showed that the baseline rate of histological high-grade squamous intraepithelial lesion (HSIL) diagnosis after HSIL cytology is high and increases with further examinations over the course of 12 months [25].

Persistence of HPV infection after treatment is probably one of the most important factors predisposing patients to the persistence or recurrence of cervical lesions. Other factors are also involved in the risk, such as the lesion size, residual disease in the margins of excisional loop electrosurgical excision procedure (LEEP) specimens, age of the patient, parity, and immunological status [26, 27].

Although HPV vaccination has not shown therapeutic effects in patients with pre-existing infection, recent data suggest that vaccination may reduce the risk of recurrence of cervical lesions. A total of 587 vaccine and 763 placebo recipients from the FUTURE I and II studies of the quadrivalent vaccine underwent excisional procedure during follow-up. Vaccination was associated with a significant reduction of 64.9% in the risk of any subsequent HSIL high-grade disease of the cervix (20.1 to 86.3%) [28]. Similarly, post-hoc analysis of the PATRICIA trial of the bivalent vaccine showed that of the total vaccinated cohort of 18,644 women, 454 (vaccine = 190, control = 264) underwent an excisional procedure during the trial. The efficacy 60 days or more post-excision surgery for the first lesion, irrespective of HPV DNA results, was 88.2% (95% CI: 14.8, 99.7) against CIN2+. Hence, women who undergo cervical excision surgical therapy for cervical lesions after vaccination with the first-generation HPV vaccines may continue to benefit from vaccination, with a reduced risk of developing subsequent CIN2+ [29].

The first study to examine vaccination in women undergoing treatment for CIN2+ was performed at the Department of Obstetrics and Gynecology of Chonnam National University Hospital, Gwangju, Republic of Korea (CNUH), between August 2007 and July 2010 [30]. Patients aged 20–45 years who were diagnosed with CIN2+ and treated by LEEP were followed: 360 patients were vaccinated with the quadrivalent HPV vaccine, and 377 patients were followed without vaccination. Irrespective of the causal HPV type, 36 (4.9%) patients developed recurrence: 9 vaccinated patients (2.5%) versus 27 non-vaccinated patients (7.2%). Multivariate analysis showed that not being vaccinated after LEEP was an independent risk factor for recurrent CIN2+ (HR = 2.840; 95% CI 1.335–6.042; *P* < 0.01) [30].

A prospective clinical study (SPERimentazione ANti HPV Zona Apuana (SPERANZA)) was designed to evaluate whether vaccination after LEEP surgical treatment in women with high-grade CIN could reduce the risk of clinical disease relapse [31]. The quadrivalent HPV vaccination of women undergoing treatment surgical therapy for CIN2+ cervical lesions and the International Federation of Gynaecology and Obstetrics (FIGO) stage IA1 cervical cancer reduced the risk of recurrent disease by 80%, suggesting a role as an adjuvant beneficial role of vaccination to surgical treatment [31].

Although data concerning non-routine HPV vaccination in populations with a high risk of HPV infection and associated lesions are scarce, multidisciplinary, evidence-based consensus guidelines for HPV vaccination in high-risk populations were developed in Spain [32]. A strong recommendation for HPV vaccination was made in the following groups: human immunodeficiency virus (HIV)-infected patients aged 9–26 years; MSM aged 9–26 years; women with precancerous cervical lesions; patients with congenital bone marrow failure syndrome; women who received a solid organ transplant or haematopoietic stem cell transplantation aged 9–26 years; and patients diagnosed with recurrent respiratory papillomatosis aged 9–26 years. In the case of women with precancerous cervical lesions, the vaccine can be provided at any time but preferentially at diagnosis or before treatment. It is not recommended to perform an HPV test before vaccination because the vaccine should be provided regardless of the HPV status [32]. These guidelines were adopted by the Spanish government in July 2018.

At the Hospital Clinic Barcelona, Spain, 265 women were treated for CIN2+ between July 2013 and July 2018. All were offered vaccination: the rate of acceptance was 37% when the vaccine was not funded (HPV vaccine after treatment was funded in July 2017) 75% when women were offered free vaccination 1–12 months after treatment, and 84% when women were immediately referred for free-of-cost vaccination after diagnosis [33]. Persistent or recurrent disease was diagnosed in 5/153 (3.3%) of the vaccinated women, whereas 12/112 (10.7%) of the women who rejected HPV vaccination were diagnosed with persistent or recurrent disease (*p* = 0.015). The protective effect of the HPV vaccine was particularly clear in women who had no disease (negative HPV test, negative Pap test, and, when performed, a negative biopsy) in the first post -excisional treatment examination at 6 months: none of the vaccinated women who were free of disease in the first post conization examination developed HSIL during follow-up, confirming that vaccination prevented the acquisition of new HPV infections after treatment. The women who had persistent low-grade squamous intraepithelial lesion (LSIL)//HPV infection in the first post-excision conization control also tended to have lower rates of persistent/recurrent HSIL at the end of follow-up when vaccinated, although the differences were not statistically significant (6.9% vs. 14.0%, *p* = 0.173 in vaccinated and non-vaccinated patients, respectively).

While vaccination-induced antibodies prevented new HPV infections in older women, it is unclear whether they also prevented the reactivation of latent, previously acquired infections, as may occur due to immune senescence in older age or specific immunosuppression. Also, the role of reactivation in clinically relevant cervical lesions needs further research.

### HPV vaccination in relation to conization: a Danish nationwide study

**Fig. 1:**
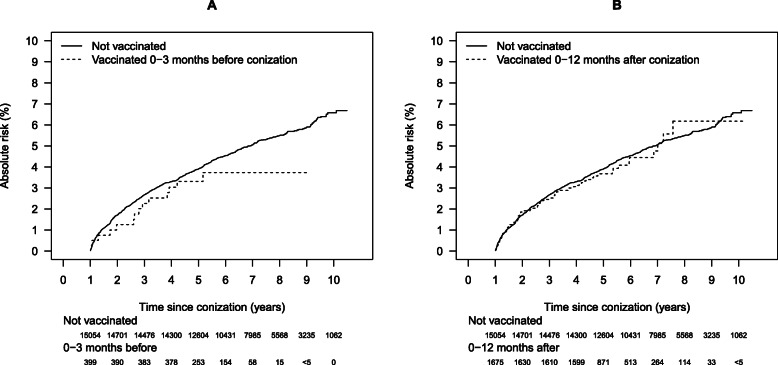
Absolute risk of CIN2+ by follow-up time according to vaccination status . Absolute risk of cervical intraepithelial neoplasia grade 2 or worse during follow-up for women not vaccinated in relation to conization and women vaccinated 0–3 months before (**a**) and 0–12 months after conization (**b**). Follow-up starts 1 year after conization. The number of women at risk is given in the risk-tables below each panel. Source: Sand, F.L., Kjaer, S.K., Frederiksen, K. and Dehlendorff, C. (2020), Risk of cervical intraepithelial neoplasia grade 2 or worse after conization in relation to HPV vaccination status. Int. J. Cancer, 147: 641–647(10.1002/ijc.32752)

In Denmark, all citizens have a personal identification number, which is used universally in society, making it possible to perform population-based surveillance studies with virtually no loss to follow-up. This study linked data from the Pathology Data Bank to those in the HPV vaccination register, leading to a study population of three groups of women treated for CIN3: 15,054 unvaccinated women, 399 women vaccinated up to 3 months before treatment, and 1675 women vaccinated up to 12 months after treatment [34]. These women were followed up for the development of CIN2+. While there was little difference between the absolute risk of CIN2+ between the unvaccinated and the group vaccinated after treatment, the risk was consistently lower for women vaccinated before treatment, and the risk diverged 5 years after treatment (Fig. 1). However, this decreased HR (0.77; 0.45–1.32) was not statistically significant.

### Transmission reduction and prevention with HPV vaccination (TRAP-HPV) study

To study the effect of vaccination on HPV transmission within stable couples, data from the HPV Infection and Transmission among Couples through Heterosexual activity (HITCH) study [35], a prospective cohort study of heterosexual couples (women ages 18–24 years) in Montreal from 2005 to 2013 were used. Among 497 couples, 12, 16, and 35 women received 1, 2, or 3 vaccination doses at baseline, respectively, with a median age at vaccination of 18 years. Most of these women (92%) had their first coitus before HPV vaccination. At baseline, partner concordance of persistent vaccine-type (VT) infections was lower in vaccinated women than in unvaccinated women [adjusted odds ratio (aOR) = 0.10; 95% CI 0.01–0.65] but not for non-VT infections (aOR = 1.00; 95% CI 0.44–2.29) [36]. The incidence of persistent VT infections in women was inversely associated with the vaccination status at baseline (adjusted HR = 0.12; 95% CI 0.03–0.47). Likewise, male partners of vaccinated women had a lower incidence of VT infections (aOR = 0.22; 95% CI 0.05–0.95). Finally, vaccinated women with VT infections had significantly lower viral loads than unvaccinated women (*P* = 0.001) [36]. These data show that the vaccination of sexually active women significantly reduced the transmission of VT HPV types in heterosexual couples.

To determine the efficacy of an HPV vaccine in reducing the transmission of genital and oral HPV infection to sexually active heterosexual partners of HPV-vaccinated individuals, a randomized clinical trial was set up (NCT01824537) [37]. The trial has a 2 × 2 factorial design, vaccinating both male and female partners with 9-valent HPV vaccine Gardasil 9, both with a placebo (hepatitis A vaccine), males with an HPV vaccine and females with a placebo, and vice versa, aiming for a sample size of 500 couples. Recruitment has been ongoing since January 2014, but the target number has been hard to reach. This is the first RCT to investigate HPV transmission reduction via vaccination within couples, which could provide empirically derived estimates for health economic models and mathematical models predicting herd immunity.

### Predicting cohort-specific cervical cancer incidence from population-based HPV prevalence surveys

The direct assessment of the time lag between HPV infection acquisition and cervical cancer occurrence is unethical. The uncertainty about the time lag may affect the validity of the cervical cancer risk estimates and of the expected impact of preventive measures.

Therefore, an attempt was made to estimate cervical cancer incidence rates in hr-HPV-positive women as a function of the time lag between a hr-HPV prevalence measurement (exposure) and cervical cancer detection (outcome). Based on this analysis, it may be possible to make longitudinal and cohort-specific projections of cervical cancer incidence from age-specific hr-HPV prevalence data. An additive Poisson regression model without an intercept term was used to comply with the restriction that cervical cancer rates should be zero if the HPV prevalence is zero. Using the average age at sexual debut, by birth cohort and by location, cervical cancer incidence projections could be made. These projections flattened from age 35 onwards. Moreover, the impact of the mean age at sexual debut was strongest in younger age groups.

The effect of hr-HPV prevalence on annual cervical cancer incidence among HPV+ women increased with the age at HPV detection and the time lag between hr-HPV prevalence and cancer incidence assessment. Furthermore, below age 35, cervical cancer risk changed based on the average age at first sexual encounter, which is a proxy for the HPV infection duration. Both findings are consistent with the current biological model of HPV progression to cervical cancer. This allows short-term predictions of the annual cancer incidence from HPV prevalence data (which are relatively easy to collect) in countries without cancer registration.

### Overview of health economics models for HPV vaccination in middle-aged adults

The United States Food and Drug Administration (FDA) approved expanding the age indication for the 9-valent HPV vaccine to age 45 in October 2018. Therefore, there is a need to perform cost-effectiveness analyses of middle-aged adult vaccination, more specifically, the cost-effectiveness of HPV vaccination for females (and possibly males) between ages 27 and 45. Five models were used to inform adult HPV vaccination policy decisions in the US: the HPV-ADVISE model (Laval University/ Centers for Disease Control and Prevention (CDC); a simplified model (CDC); the Merck model; the Harvard model; and the Policy1-Cervix model (Cancer Council New South Wales). All five models include a wide range of health outcomes (cervical precancers; cervical, anal, vaginal, vulvar, penile, and oropharyngeal cancers; and anogenital warts), all account for herd effects and examine a long time period (~ 100 years).

However, the models differ in structure, calibration, cervical cancer screening assumptions,

vaccine uptake assumptions for middle-aged adults, the natural history of HPV parameters, HPV transmission dynamics, and cost and quality of life assumptions.

Routine vaccination at ages 11 or 12 years with catch-up vaccination through age 26 years for females and age 21 years for males was shown to be cost-effective in all models, ranging from cost savings in the HPV-ADVISE model to $34,600 per quality-adjusted life year (QALY) gained versus no vaccination in the Harvard model. In June 2019, the Advisory Committee on Immunization Practices recommended to extend catch-up vaccination for males through age 26 year, making it the same as the existing recommendation for women.

**Fig. 2:**
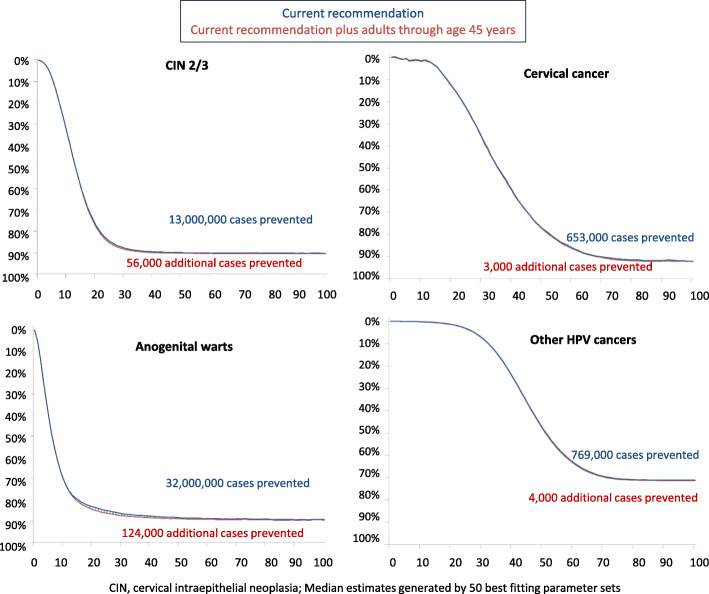
Estimated impact of adult HPV vaccination [38]. Source: Brisson ACIP, February 2019, CIN = cervical intraepithelial neoplasia; median estimates generated by 50 best-fitting parameter sets

The vaccination of adults up to the age of 45 would prevent an additional 56,000 cases of CIN2/3 compared to the 13,000,000 cases prevented by the current recommendation (0.43%). Similarly, an additional 3000 cases of cervical cancer compared to the 653,000 cases prevented by the current recommendation (0.46%); an additional 124,000 cases of anogenital warts compared to the 32,000,000 cases prevented by the current recommendation (0.39%); and an additional 4000 cases of other HPV-related cancers compared to the 769,000 cases prevented by the current recommendation (0.52%) (Fig. 2) [38]. The cost per QALY gained, based on the 5 models, would range from $117,500 to $1,471,000 when extending the age to 45. Assuming faster progression and lower natural immunity, these figures would come down. However, for the HPV-ADVISE model, they would remain over $1,000,000 per QALY gained, while the figures would only become worse assuming slower progression and higher natural immunity.

In summary, the cost per QALY gained by the current vaccination programme is below $35,000 in all models and even lead to cost savings in the HPV-ADVISE model, whereas adult vaccination is much less cost-effective. Notable differences in cost-effectiveness estimates exist across models, likely due to uncertainties in HPV natural history and transmission dynamics, which preclude a precise estimate of the cost-effectiveness of vaccination in adults. Furthermore, the results were more consistent when standardizing health economic assumptions and assumptions regarding deaths due to undiagnosed cancer. In the context of the existing programme, vaccinating adults over 26 years of age would produce relatively small additional health benefits, with the number needed to vaccinate to prevent one case of disease being approximately 40 times higher.

## HPV vaccination in high-risk groups

### Directionality of HPV infection transmission in heterosexual couples: a systematic review and meta-analysis

In individual-based studies, researchers can determine HPV prevalence and incidence rates by conducting multiple follow-up visits on the same study subjects. Couple-based research could permit the examination of transmission dynamics between partners, augmenting the information collected in individual-based HPV transmission research.

The EUropean Research Organisation on Genital Infection and Neoplasia (EUROGIN) 2014 Roadmap provided a descriptive overview of five heterosexual couple-based longitudinal studies in its discourse of HPV transmission rates, suggesting greater rates of female-to-male (F-M) than male-to-female (M-F) transmission. A systematic review and meta-analysis were performed to assess the evidence for the differential transmission rate hypothesis in couple-based studies in regard to genital-to-genital HPV transmission [39]. Seven longitudinal studies on heterosexual couples in whom genital samples were collected were included. Several different HPV detection methods were used, which may have impacted the study findings.

The overall rate difference was 0.61 infections per 100 person-months (95% CI -0.27-1.49), indicating that F-M transmission is higher, although the result was not significant, and substantial statistical heterogeneity existed (I^2^ = 75%). Excluding two potential outliers did not alter the rates significantly; F-M transmission was still favoured. However, these findings must be interpreted with caution due to the presence of substantial statistical heterogeneity.

### HPV vaccination for sex workers: finding a balance between the pros and cons

A study including 304 female sex workers (SWs) in Amsterdam, the Netherlands, showed that the vaginal and anal hr-HPV prevalence were 46 and 55%, respectively, whereas anti-HPV seropositivity, against a least one of the high risk types included in the Gardasil 9 vaccine, was 37% [40]. This led to the hypothesis that HPV vaccination of SWs, preferably at the initiation of sex working, may be a useful prevention method against hr-HPV infection, disease and potentially transmission. Therefore, determinants of their intention to become vaccinated against HPV were explored. Vaccination intention was high but decreased significantly when vaccination would require out-of-pocket payment [41].

There are several reasons to offer HPV vaccinations to SWs [42]: SWs are at a high risk for HPV infection; they are unlikely to have been vaccinated in the past; the immunogenicity of vaccines is excellent in all women (even if previously exposed); women with HPV disease may still benefit from vaccination; SWs may not have been exposed to all HPV types covered by HPV vaccines; and vaccination may reduce the transmission of HPV to clients. On the other hand, there are also reasons not to offer the vaccine to SWs: the current vaccines are prophylactic and have not been shown to be therapeutic; it is difficult to establish a woman’s previous HPV status, as more women may have been infected than a DNA test or serology shows; it is difficult to establish whether HPV was cleared or has gone into latency; finally, vaccinating after sexual debut may not offer protection against CIN2+ or anal intraepithelial neoplasia (AIN2+)+) [7, 43].

Although HPV vaccination should be offered to all girls prior to sexual debut, studies are needed to directly or indirectly establish the effectiveness of vaccinating SWs. Although RCTs generally provide the best answer, these are not realistic in the current setting. Because vaccines are safe, a benefit/risk balance is not needed, and a cost-benefit balance will suffice.

### Baseline HPV prevalence in rectal swabs from men attending a sexual health clinic in Scotland: assessing the potential impact of a selective HPV vaccination programme for MSM

As MSM are at a high risk of HPV infection and associated disease and have little to no benefit from the girls-only vaccination programme, a vaccination programme was recommended for MSM attending sexual health clinics in Scotland [44]. The programme was implemented in July 2017 for MSM under 45 years of age as well as prisoners and transgender women. The uptake of the HPV vaccine in Scotland for MSM is approximately 65% for the first dose, but completion of the schedule is much lower. Uptake is highest in the 20–29 age group. To examine the impact on disease, data were extracted from the national sexual health database on the number of genital wart treatment prescriptions. While declines could be seen in the numbers of prescriptions for both women and in heterosexual men, this was not the case for MSM. Similarly, rectal swabs were used to assess the impact of vaccination on HPV prevalence, comparing prevalence in samples taken before (*n* = 1209) and after (*n* = 1235) the introduction of the program. This showed a slight and non-significant increase in HPV-6/11 types 6 and 11 and a significant decrease in HPV-16/18 types 16 and 18 from 37.9 to 31.8% (odds ratio (OR) 0.76, *p* = 0.0014). Although it is an indication of an early vaccine effect, confirmation will be necessary. Moreover, further work is underway to link the vaccine status to genital wart treatment prescribing data, HPV prevalence data, and, in the long term, HPV-associated cancers.

## Limitations and elimination goals

### Towards cervical cancer elimination: the context of HPV vaccination

**Fig. 3:**
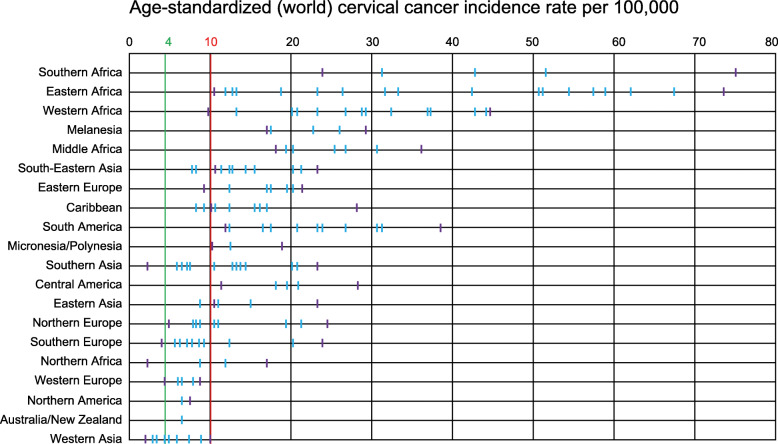
Variability in cervical cancer incidence rates by world region. Source: WHO IVB Database, October 2019. Region: country with lowest incidence – country with highest incidence. Southern Africa: Namibia – Swaziland; Eastern Africa: Fr. La Reunion – Malawi, Western Africa: Niger – Guinea; Melanesia: New Caledonia – Papua New Guinea; Middle Africa: Gabon – Angola; South-Eastern Asia: Malaysia – Indonesia; Eastern Europe: Poland – Moldavia; Caribbean: Puerto Rico – Jamaica; South America: Brazil – Bolivia; Micronesia/Polynesia: Fr. Polynesia – Guam; Southern Asia: Iran – Maldives; Central America: Mexico – Belize; Eastern Asia: China – Mongolia; Northern Europe: Finland – Latvia; Southern Europe: Macedonia – Bosnia Herzegovina; Northern Africa: Tunisia – Morocco; Western Europe: Switzerland – Belgium; Northern America: Canada – USA; Australia/New Zealand: Australia; Western Asia: Qatar - Georgia

There is large global variability in cervical cancer incidence rates, with exceptionally high rates in the African region. However, even in regions with high incidence rates, exceptions exist, such as La Reunion in Eastern Africa and Niger in Western Africa, showing rates of approximately 10/100,000 women, which is well below the rates in some countries in northern, southern and eastern Europe (Fig. 3).

Three HPV models, the Policy 1 model, the Harvard model and the HPV-ADVISE model, as well as an HIV model, the Spectrum model, were selected, as they are dynamic models that include vaccination, screening and treatment. These models were used to investigate three scenarios. Scenario 1 is based on girls-only vaccination, with no change in screening compared to the current situation. Scenario 2 is based on girls-only vaccination, with once-in-a-lifetime screening at 35 years of age. Scenario 3 is based on girls-only vaccination, with two lifetime screens at 35 and 45 years of age. All scenarios are based on HPV-based screening, 100% treatment efficacy, 10% loss to follow-up for screening and 90% coverage of 9 to 14-year olds, the lifelong duration of immunity, and 100% efficacy for 7 h-HPV types for the vaccine. While all scenarios indicated that low-income countries (LICs) will go below the 10/100,000 threshold, the 4/100,000 threshold was only reliably reached by scenario 3.

Looking at the cervical cancer incidence, the addition of boys to the vaccination programme has shown little impact in India, Vietnam and Nigeria and some impact in Uganda, while catch-up vaccination up to the age of 25 has had no impact in any of these four countries.

These data have led to the development of a global strategy directed towards the elimination of cervical cancer, aiming for all countries to get below the incidence level of 4 cases/100,000 women. The 2030 targets set to achieve this goal are as follows: 90% of girls fully vaccinated at the age of 15; 70% of women screened with a high-precision HPV-based test at the ages of 35 and 45; and treatment and care for 90% of women with identified cervical disease.

Although 50% of all countries in the world have introduced the HPV vaccine, this only covers approximately 30% of all girls globally. Furthermore, while 86% of HIC and 58% of upper-middle-income countries (MICs) are covered, only 33% of lower MICs and 16% of LICs are covered, which are the countries where the burden is the highest.

There is an insufficient vaccine supply to meet the overall demand of the Global Alliance for Vaccines and Immunisation (GAVI) countries, although all planned GAVI-supported HPV vaccine introductions are moving ahead with routine cohorts, without room for multi-age cohorts (catch-up). At least one MIC had to postpone introduction in 2019 due to the lack of supply. The supplies are expected to slowly grow over the next 1–3 years, followed by quicker growth in the mid- to long-term. Several scenarios have been used to compare dose requirements, including a 2-dose and a 1-dose schedule, with or without catch-up vaccination, and extended schedules with the second dose after 3 or 5 years. At a base supply level, all scenarios experience a shortage in the short term, with the 3-year extended interval being least affected, but supplies will be adequate in the mid- to long-term time period. However, in the case of low supply, only 1-dose schedules will have adequate supplies in the mid- to long-term, with the 3-year extended interval as a possible alternative. The introduction of catch-up programmes, including the vaccination of adults, may lead to postponed introductions in 1–27 countries in the short term, leaving 45,000–143,000 girls/women vulnerable to cervical cancer. Some HICs have already introduced gender-neutral vaccination, which requires 9 million (M) doses or 18% of the global demand. If other HICs add boys to the programme, this will require another 4 million doses, which might mean that introduction is delayed in 12 LMICs. This led to the Strategic Advisory Group of Experts on Immunization (SAGE) recommendation that countries should temporarily postpone the implementation of gender-neutral, older-age group (> 15 years) and multi-age cohort HPV vaccination strategies until all countries have access to the HPV vaccine. This will significantly relieve supply constraints in the short term and enable the allocation of doses to high-burden countries currently planning to introduce this vaccine. It is important to note that the above estimates were made prior to the coronavirus pandemic and so could not consider any reduction in vaccine uptake that might have resulted from interruptions in school and clinic-based programs.

## Plenary discussion

Several topics were discussed as part of the plenary sessions in the meeting.

### Could cervical smear (PAP) taking increase the risk of HPV infections?

First, it was stressed that this is a purely scientific issue and not a public announcement. The value of cervical cancer screening is not in question: there is ample evidence that screening has led to a decrease in the incidence of cervical cancer as well as associated mortality [45–47]. However, this does not preclude testing the hypothesis that the rising incidence in adenocarcinoma could be related to more aggressive sampling to include endocervical cells in the sample. Nevertheless, alternative explanations for this increase were offered, including changes in sexual behaviour. The progressive lowering of the age of coitarche would lead to early HPV infections, which could also be a reason for the increase in adenocarcinoma, as the immature cervix during adolescence may be more susceptible to persistent HPV infection. The combination of sampling with the use of carrageenan may significantly reduce the risk of infection. Multiple trials are ongoing to confirm this. Alternatively, atraumatic self-sampling has been extensively evaluated for HPV DNA-based testing and is being considered for screening programs [48].

### The rationale for vaccinating middle-aged adults

The key question is what the risk of developing cancer by the age of HPV infection acquisition is. What kind of studies need to be done to find this answer? Can the ongoing trials be used to develop estimates for modeling, providing a distribution of the risk by age of infection?

Alternatively, trials are needed to compare the risk of development of precancerous lesions after vaccination in middle-aged adults. Although it is known that most infections occur soon after the start of sexual debut activity, 15–20% of infections occur after the mid-20s.

Although the impact of hormonal changes during pregnancy on HPV infection is known, this is not the case for menopause. A number of observational studies have shown a second peak in HPV prevalence in older age [49].

### The ethics of vaccination

There is a strong consensus it is unethical not to vaccinate girls in general and in particular in LMICs: these are the countries with the highest burden and where establishing and performing effective cervical cancer screening programs, as well as treatment, is much more complicated. Hence, the vaccine is their best hope.

However, not giving boys a chance to be vaccinated also has ethical implications.

Data from a number of countries, including the US, show that the oropharyngeal cancer incidence is increasing [50–55], and the majority of this cancer in some countries is HPV-related, whereas other head and neck cancers are not necessarily HPV-related, and this fraction, mostly caused by tobacco and alcohol, is decreasing. In some countries with effective cervical cancer screening, oropharyngeal cancer incidence has surpassed cervical cancer incidence, and oropharyngeal cancer occurs predominantly in males. However, it cannot be ruled out that most oropharyngeal cancers occurring in males could be prevented by the vaccination of females. Currently, there are only limited data on impact on infections available. However, in June 2020 the FDA approved: “Prevention of oropharyngeal and other head and neck cancers caused by the HPV types targeted by Gardasil 9” to the vaccine’s indications.

### Vaccine supply

The pharmaceutical industry is trying to increase the HPV vaccine supplies: Merck is increasing vaccine production, GlaxoSmithKline Biologicals (GSK) is re-opening its vaccine-producing factory, and the Innovax bivalent vaccine has now been licensed in China. While important, these developments would not be enough to solve the immediate problem of shortages. The shortage is foreseen to be resolved by 2023/2024.

Intradermal immunization may be an alternative strategy to evaluate. It has been done for influenza at 1/10th of the dose. This solution has also been contemplated for polio and rabies vaccines. In some regions, e.g., eastern Europe, intradermal delivery is already quite common. However, proper intradermal delivery is more difficult, and switching from intramuscular to intradermal delivery will take time and requires effort.

### Lessons learned

- Older cohorts can be vaccinated for different purposes: 1) to benefit the community by reducing transmission or healthcare costs; 2) to benefit the individual, although the latter might be limited in older cohorts based on the studies reviewed during the meeting.
- If vaccinating entire older cohorts is not possible considering the higher costs or shortage of vaccine supply, the focus could be on high-risk groups, such as MSM or HIV+ women.
- Evidence is accumulating that the vaccination of women with CIN2+ before or after treatment reduces disease recurrence of disease. Provision of the vaccine treatment should be considered, regardless of age.
- Two sites are available to block HPV infection by antibodies: 1) the binding of the virus to the basement membrane and 2) the binding of the virus to L1 binding sites on keratinocytes for transport into the cell.
- To protect against HPV infection, low levels of antibodies may be sufficient, but maybe high avidity is necessary.
- Given the preventive effect of carrageenan, its use as a standard lubricant during pelvic exams should be considered. Three trials using carrageenan are currently underway; preliminary results show that it is 40% efficacious as a general use vaginal gel against incident HPV infection.
- The priority for HPV vaccination lies with the target groups: to achieve high coverage in girls and boys between 9 and 14 years of age first.
- The vaccination of boys is not just focused on cervical cancer prevention; it is also directed at several HPV-related cancers that also occur in men. For instance, in many HICs, there has been a steady increase in the number of HPV-related oropharyngeal cancers, with the HPV-related cancer burden in men approaching that in women. Furthermore, gender-neutral vaccination may be a way to increase protection against all HPV-associated cancers in countries with lower vaccination coverage in females.
- To date, the goals and policies for HPV vaccination have been focused on benefits for vaccinated individuals. It may be necessary to think more broadly: by blocking transmission, a rapid drop in HPV prevalence may be achieved.
- It may be too early to think about the elimination of cervical cancer. We should aim to increase HPV vaccination coverage in countries with the highest cervical cancer incidence first, even if formal elimination will only be possible if vaccination is combined with screening and treatment, which may be too costly for many LMICs.
- The impact of a pause/delay in the use of a valuable vaccine should not be underestimated. Anti-vaccine messaging can have a large negative effect on vaccine acceptance in both HICs and LMICs; therefore, these must be strongly countered.
- If the goal truly is to eliminate cervical cancer as a major public health problem, we need to immunize whole populations, analogous to the polio eradication effort. However, the eradication of cervical cancer may not be achieved: a rate of less than 4/100,000 women is not eradication of the cancer or the underlying HPV infections.

We have to think globally, as the need for vaccines in HICs, MICs, and LICs is the same, with younger cohorts as a priority. However, we should not overlook the potential benefits such as reduced costs of screening programs it can offer in the future.

Many evidence gaps exist, making further studies necessary. The following studies were suggested:

- Now that more data are available, revisit the efficacy of HPV vaccination in older women
- Investigate the impact of HPV vaccination on reducing transmission with tissue culture experiments, demonstrating that shed virus can be neutralized or with RCTs showing a reduced risk of transmission
- Develop more sensitive assays to detect mucosal anti-HPV antibodies and use these assays in combination with standardized sample collection to increase comparability between studies
- Investigate the risk of progress to cervical cancer development after primary HPV acquisition at different ages
- Develop RCTs to investigate the collection of cytology samples in the presence or absence of carrageenan
- Based on large data sets, including HPV genotyping on cytology samples, after a change in sampling device: look for an effect on glandular disease before and after the new device
- Identify a small, not previously vaccinated population, preferably on an island, and give everybody a single dose of vaccine. This should have a profound impact on transmission. At the same time, it may be possible to assess the potential for elimination
- Given the shortage in vaccine supply, intradermal immunization to save VLPs should be considered, so that a delay in vaccination in LMICs may be avoided. While switching to intradermal administration will take time and effort, it may be useful for other antigens at a later point (e.g., pandemic influenza)
- Studies will have to be conducted to look at the effectiveness of the vaccination of SWs, both for SWs themselves (disease prevention) and for society, by reducing transmission from a potential reservoir
- Further studies are needed to substantiate the evidence that vaccination after excision of pre-cancerous lesions helps to reduce the persistence and/or recurrence of the lesions.

## Conclusion

Based on the data presented and the discussion that took place, it can be concluded that it is premature to introduce routine vaccination of adults, as the information is incomplete and many further studies are needed (see above) to fill the knowledge gaps.

Given the limited HPV vaccine supply in the short term, the vaccination of adults should not consume vaccine doses. Vaccination of younger age cohorts remains the priority, especially in LMICs, as these are the countries with the highest burden of HPV-associated cancers.

## Data Availability

All the presentations of the meeting report are published on the website (www.hpvboard.org) after speakers’ approval.

## Abbreviations

HPV–PCB: HPV Prevention and Control Board
HPV: Human Papillomavirus
SWOT: Strengths, Weaknesses, Opportunities, and Threats
HIC: High Income Countries
NIP: National Immunization Programs
LMICs: Low- and Middle-Income Countries
IgG: Immunoglobulin G
IgA: Immunoglobulin A
VLPs: Virus like particles
CVS: Cervicovaginal secretions
CIN: Cervical Squamous Intraepithelial Neoplasia
MSM: Men who have sex with me
LEEP: Loop electrosurgical excision procedure
CNUH: Chonnam National University Hospital, Gwangju, Republic of Korea
HSIL: High grade squamous intraepithelial lesion
HR: High Risk
CI: Confidence Interval
VT: Vaccine-type
hr-HPV: High risk Human papillomavirus
US: United States
FDA: Food and Drug Administration
QALY: Quality-adjusted life-year
CDC: Centers for Disease Control and Prevention
EUROGIN: EUropean Research Organization on Genital Infection and Neoplasia
FIGO: International Federation of Gynaecology and Obstetrics
F-M: Female to Male
M-F: Male to Female
LICs: Low income countries
GSK: GlaxoSmithKline Biologicals
SWs: Sex Workers
GAVI: Global Alliance for Vaccines and Immunizations
